# Respiratory insufficiency, feeding issues and length of stay in 33–36 weeks post-menstrual age infants

**DOI:** 10.1038/s41390-025-04411-4

**Published:** 2025-09-27

**Authors:** Anjeline Bukhari, Zahraa Dawoud, Selphee Tang, Michelle Matthews, Kamran Yusuf, Shabih U. Hasan

**Affiliations:** 1 Departments of Pediatrics, University of Calgary, Calgary, Canada; 2 Obstetrics and Gynecology, Cumming School of Medicine, Alberta Children’s Hospital Research Institute, University of Calgary, Calgary, Canada

## Abstract

**Background/Aims:**

Limited post-menstrual age (PMA) stratified data are available for the morbidities and length of stay (LOS) for the largest group of preterm infants. We investigated the incidence, types and interactions of morbidities that prolong the LOS at 33–36 weeks PMA.

**Methods:**

Electronic and bedside charts of 1209 infants were visually reviewed. Major outcomes included respiratory support, achievement of gavage-free feeding and maternal/infant variables associated with shorter/longer than Median LOS. Fisher’s exact tests/ANOVA/logistic regression were used for statistical analyses.

**Results:**

The Median (IQR, Range) of the LOS were distinct at each and even within PMA between 33 and 36 weeks (*P* < 0.001). 63% of infants born at 33-weeks received respiratory support vs. 46, 39 and 7% born at 34-, 35- and 36-weeks, respectively (*P* < 0.001). Multiple births, BW within a given PMA, SGA status, respiratory support, RDS, delayed gavage-free feeds and birthplace were associated with longer than Median LOS at each PMA (*P* ≤ 0.04). Achievement of gavage-free feeding was consistently the main determinant of early discharge home across all PMAs (*P* < 0.001).

**Conclusions:**

Our newer approach in identifying relationship among morbidities in infant born at 33–36 weeks PMA fills important knowledge gaps. These data will facilitate evidence-based clinical care, educational-needs, health care resource planning and parental counseling.

**Impact:**

- Either grouped and/or fragmented data are available for morbidities in infants born between 33 and 36 weeks post-menstrual age (PMA), which represents >80% of all preterm infants.
- We demonstrate that respiratory insufficiency, type of respiratory support, delayed gavage-free feedings and length of stay (LOS) are inter-dependent and PMA-specific.
- Using a novel approach, we provide new significant data that identify clinical variables, associated with shorter and longer than Median LOS at each and even within a given PMA.
- Comprehensive analysis of morbidities suggests that preterm infants should neither be grouped, nor PMA alone be used for discharge planning and parental counseling.

## Introduction

Globally over 15 million infants are born preterm each year, defined as those born <37 weeks post menstrual age (PMA).^1^ Most neonatal research has focused on extremely preterm infants born at <28 weeks PMA^2–5^ who comprise less than 15% of the preterm population. Over 84% of preterm infants born between 32 and 36 weeks PMA and over 70% between 34 and 36 weeks PMA^6^ are at an increased risk of short- and long-term morbidities but remain understudied.^7–13^ Furthermore, this largest group of preterm infants significantly increases the Neonatal Intensive Care Unit (NICU) bed occupancy^14^ and economic cost to health care system.^15–18^ Limited PMA-specific data are available on the prevalence and/or interaction between respiratory support, oral feeding milestones and length of hospital stay (LOS).^19,20^ Furthermore, data or guidelines for feeding late preterm infants are sparse^21^ or remarkably absent.^22^ Unavailability of PMA-specific morbidities and LOS pose challenges to knowledge-based clinical care, education of health care providers, resource allocation and parental counseling.^23–27^ Parents of preterm infants have identified the need to be counseled about the reason (s) of admission, expected neonatal problems, postnatal course, and the LOS.^28^ Availability of such PMA-specific morbidities are vital for decreasing psychological distress and fostering a trusting relationship between parents and health care providers, the importance of which cannot be over-emphasized.^1,28–30^

Major limitations of the studies performed to date include pooled analysis of morbidities for the entire cohorts of moderate (32–33 weeks) and late (34–36 weeks) preterm infants^31^ and/or comparisons made between late preterm and term infants.^19,32–34^ One would anticipate that neonatal morbidities and the (LOS) would be markedly different between and within these groups.^35^ Furthermore, the interaction of multiple clinical variables has not been investigated, which is vital for making informed decisions. In one study, the level of neonatal care was not available with certainty and extrapolated from the American Academy of Pediatric definitions and the LOS did not assess the impact of respiratory support and ability to feed orally were not taken into consideration.^36^ Hence, such information cannot be considered applicable to everyday practice. Moreover, stratified data dating back to 2–4 decades^37,38^ may not reflect current neonatal practice especially the use of non-invasive respiratory support and initiation of early enteral/oral feeds. Finally, studies have investigated morbidities in a piecemeal fashion, thus not encompassing the overall morbidity.^19,20,33,35,39–42^ Thus, much-needed contemporary data for respiratory support, achievement of oral feeding skills, and LOS for the late preterm infants remain unclear.^22^

The *Specific Aims* of the current study were to (1) investigate the incidence, type, and duration of respiratory support at each PMA between 33 and 36 weeks PMA, (2) assess the impact of maternal and infant characteristics on neonatal morbidities and LOS and (3) to elucidate the specific clinical variables, not in the causal pathway that delay infants’ discharge home. We hypothesize that the neonatal morbidities would be markedly different between and within these PMA, and the LOS would be dependent on neonatal morbidities in an interdependent fashion and achievement of gavage-free oral feeding.

## Methods

### Ethics and study population

This study was approved by the Conjoint Health Research Ethics Board of the University of Calgary and conducted in conformity with the principles and regulations of Helsinki Declaration. We have followed the STROBE guidelines. The study was performed at the regional tertiary care NICU at Foothills Medical Centre, affiliated with the University of Calgary, Canada.

The study included all preterm infants born and admitted to the NICU between 33^0^ and 36^6^ weeks PMA, from April 1, 2016 to March 31, 2021. Data extraction, validation, and analyses were updated in August 2024. The rationale for including 33- through 36-weeks PMA was that the Canadian Neonatal Network reports mortality and morbidity data on infants born at <33 weeks PMA and scarce feeding^31^ and discharge home data are reported in the literature.

Our policy to admit all infants born at 33–35 weeks PMA is based on several studies^43–48^ which demonstrated higher rates of re-admissions even at 35 weeks PMA^48,49^ and in-hospital and/or post-discharge morbidities.^43,44,48^ Furthermore, early discharge of late preterm infants does not always translate into cost savings.^50^ The reasons for readmissions after early discharge include hyperbilirubinemia, feeding difficulties, cardiorespiratory events, and may be a traumatic experience for the parents.^49^ Although rare, preterm infants born at 33-36 weeks are at more than twofold risk of sudden unexpected infant death syndrome^51^ and is considered an unacceptable event.^52^ As suck-swallow-breathe repertoire does not mature until 35 weeks PMA,^53^ early discharge could lead to feeding difficulties and failure to thrive. Thus, extra caution is warranted prior to discharging infants home, who exhibited cardiorespiratory instability during the postnatal period. The admission criteria at 36 weeks in our center include respiratory distress, need for supplemental oxygen, birth weight of <2250 g, small for gestational age at <3rd centile, potential/suspected sepsis necessitating investigations, cardiorespiratory monitoring and antimicrobial administration, hypoglycemia not responding to oral feeding or dextrose (gel) administration, need for gavage feeds and congenital anomalies requiring observation and/or investigations. Based on these criteria, 43.7 ± 1.95% (Mean ± SD) of infants born at 36 weeks were admitted to the NICU. Outborn infants were defined as those born outside the tertiary care center and comprised 5.8% of all infants. The indication for transfer included birth weight <1500 g, respiratory distress necessitating mechanical ventilation and anomalies requiring investigations.

**Fig. 1:**
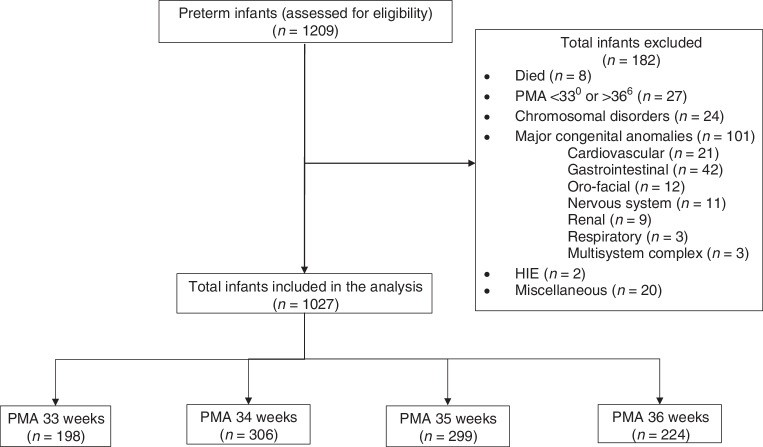
Flow diagram of study population. The total number of infants assessed for eligibility (*n* = 1209), excluded (*n* = 182) and those, who met the inclusion criteria (1027).

The exclusion criteria comprised PMA age of <33^0^ or >36^6^ presence of major congenital anomalies, chromosomal disorders, and conditions that might prevent the infant from sucking feeds orally and/or achieving gavage-free full oral feeds and death before discharge (Fig. 1). Such conditions included neuromuscular disorders, fetal alcohol spectrum disorder, neonatal abstinence syndrome, or who were kept nil-per-orem (NPO) for suspected sepsis and/or necrotizing enterocolitis (Fig. 1).

### Study design

Maternal variables were stratified into 33-, 34-, 35- and 36- completed weeks PMA and included, age, gravidity/parity, diabetes mellitus, gestational diabetes mellitus, pre-eclampsia, gestational hypertension, pre-existing hypertension, use of assisted reproductive technologies, antenatal glucocorticoids and number of doses administered, mode of delivery, maternal fever, multiple birth, and history of cigarette smoking and drug use. Gestational hypertension was defined as systolic blood pressure >140 mmHg and diastolic blood pressure >90 mmHg. Preeclampsia was defined as recently broadened by the International Federation of Gynecology and Obstetrics (FIGO) initiative on pre-eclampsia.^54^

Infant characteristics, also stratified according to the PMA, comprised of BW, sex, SGA status, resuscitation and any respiratory support and respiratory support type. Resuscitation was defined as any intervention other than drying and brief suctioning. Data were also obtained for the type and duration of mechanical ventilation, exogenous surfactant administration, continuous positive airway pressure (CPAP), humidified heated high flow via nasal cannula (HHFNC at ≥2 lpm) and low flow nasal flow at <2 lpm. In our NICU, we initiate CPAP if the infant demonstrates clinical signs of respiratory distress such as tachypnea, intercostal/subcostal indrawing and/or grunting. Depending on the level of CPAP support and infant’s clinical status, they are either weaned off to high or low flow nasal cannula or straight to room air. The criteria for endotracheal intubation included worsening clinical respiratory distress (as defined above), FiO_2_ > 0.3, pH ≤ 7.2, PCO_2_ ≥ 60 torr, one apnea per hour and/or an apnea requiring positive pressure ventilation and/or radiological findings consistent with respiratory distress syndrome (RDS). Other infant variables included umbilical cord arterial pH, the Score for Neonatal Acute Physiology (SNAP II) and SNAP with perinatal extension (SNAP PE), 5-min APGAR score of <7, outborn, RDS, patent ductus arteriosus (PDA), intraventricular hemorrhage (IVH), pneumothorax, pulmonary hypertension, apnea/bradycardia. RDS was defined as clinical features of respiratory distress, radiologic changes and respiratory acidosis on blood gas analyses. The diagnosis of PDA was based on echocardiographic findings, reported by one of the pediatric cardiologists. IVH was confirmed via cranial ultrasonic examination using Papile classification.^55^ Apneas and bradycardias were defined according to our previous study.^56^

Feeding variables included any enteral feed; breast, gavage, bottle, or a combination thereof on a given day. Using our feeding protocols, we initiate enteral/oral feeds on the day of birth at 60 ml/kg/day and advance at rates of 20–30 ml/kg/day to a maximum of 140–160 ml/kg/day. There is little variability in feeding practices as adherence to feeding protocols by the clinicians is expected at all our NICUs. Achieving full oral feeds was considered if the infant was gavage-free for all feeds. The LOS was considered the time from birth and/or admission until they were discharged home. The criteria for discharge home included absence of apneas, bradycardias and/or oxygen desaturation episodes for at least 7-days, discontinuation of respiratory support for a minimum of 48 h, SpO_2_ > 90% for >90% of the time per 24 h, normal thermoregulation in a cot and ability to feed gavage-free for 48 h or more, and body weight of over 1800 g.

### Data sources and extraction

The data for all maternal and neonatal variables were extracted using electronic health records (Sunrise Clinical Manager and the NICU database) and/or bedside paper chart. Despite the importance of enteral/oral feeding,^57^ feeding data at granular level are not readily extractable from the electronic database. Hence, we visually reviewed bedside charts of all 1027 subjects and data obtained (Fig. 1). The data collection personnel included four study team members, three collecting and verifying each data point, and the fourth resolving any interpretation challenges.

### Statistical analysis

Association between maternal and infant characteristics and feeding milestones with PMA at delivery were tested using Chi-square or Fisher’s exact test for categorical variables and ANOVA or Kruskal–Wallis test for continuous variables. For variables with a significant association with PMA at *p* < 0.05, pairwise comparisons between PMAs, with the Bonferroni correction were applied to the 6 multiple comparisons (with alpha=0.05/6 = 0.0083). For variables assessed using ANOVA, the Tukey–Kramer test was used to determine which pairwise comparisons were significantly different. The Mann–Whitney test was used to compare Median number of days to achieve full oral feeds for those receiving and not receiving respiratory support for each PMA separately.

To investigate PMA-specific characteristics associated with longer lengths of stay, infants in each PMA were grouped into shorter or longer length of stay based on whether their LOS was less than or equal to, or greater than the Median LOS. For each PMA separately, the shorter and longer length of stay groups were compared using the Chi-square or Fisher’s exact test for categorical variables, and *t*-test or Mann-Whitney U test for continuous variables.

Logistic regression was used to explore the characteristics that contributed to longer lengths of stay for each PMA separately. Candidate variables for the models were those with univariate analysis *P* < 0.25, excluding variables with zero counts in any length of stay group, and excluding feeding milestones and respiratory support as these variables are likely to be in the causal pathway between maternal/infant characteristics and length of stay. Backward elimination with a threshold of *P* < 0.05 was used to identify parsimonious models. Data were analyzed using SAS version 9.4 (SAS Institute Inc, Cary, NC). A *P*-value of ≤0.05 was considered statistically significant.

## Results

Of the 1209 preterm infants of both sexes assessed for eligibility, 1027 met the inclusion criteria. A total of 182 infants were excluded; eight died, 27 were of PMA < 33^0^ or >36^6^ and 24 had chromosomal disorders. One or more major congenital anomaly was observed in 101 infants, two had HIE and 20 had complex miscellaneous conditions. Among the 1027 participants, 198, 306, 299 and 224 were born at 33-, 34-, 35- and 36-weeks PMA, respectively (Fig. 1).

### Maternal characteristics

Maternal age was similar across the infants born at 33 through 36 weeks PMA (Table 1). The proportion of women delivering at 34 weeks PMA, in whom pregnancy was conceived using assisted reproductive technologies (ART) was almost twice as high as those delivering at 36 weeks PMA (*P* = 0.027; Table 1). Furthermore, 87% of the mothers delivering at 33 weeks received ANGC compared with 50%, 25%, and 14% at 34-, 35- and 36-weeks PMA, respectively (*P* < 0.001). The remainder of the maternal variables, listed under Study Design were similar across various PMA groups (Supplementary Table S1).

**Table 1:** Maternal and infant characteristics by post menstrual age at delivery.

	Post menstrual age (PMA)				
	33 weeks *n* = 198	34 weeks *n* = 306	35 weeks *n* = 299	36 weeks *n* = 224	*p*-value^a^
Maternal characteristics					
Age, mean ± SD	32 ± 5	33 ± 5	33 ± 5	33 ± 5	0.101
Primigravid, *n* (%)	61 (31%)	88 (29%)	88/298 (30%)	61/223 (27%)	0.884
Nulliparous, *n* (%)	111 (56%)	133 (44%)^b^	132 (44%)	97 (43%)	0.019
ART, *n* (%)	36 (18%)	62 (20%)	41 (14%)	26 (12%)^c^	0.027
Antenatal steroids, *n* (%)	171/196 (87%)	150/301 (50%)^b^	75/296 (25%)^b, c^	32 (14%)^b, c, d^	<0.001
Infant characteristics					
Birth weight, mean (SD)	1925 (380)	2185 (384)^b^	2458 (420)^b, c^	2533 (534)^b, c^	<0.001
Male, *n* (%)	94 (47%)	159 (52%)	150 (50%)	132 (59%)	0.097
SGA, *n* (%)	27 (14%)	30 (10%)	24 (8%)	62 (28%)^b, c, d^	<0.001
Outborn, *n* (%)	20 (10%)	21 (7%)	11 (4%)^b^	18 (8%)	0.036
Resuscitation, *n* (%)	33 (17%)	86 (28%)^b^	117 (39%)^b, c^	82 (37%)^b^	<0.001
Any respiratory support, *n* (%)	124 (63%)	141 (46%)^b^	118 (39%)^b^	16 (7%)^b, c, d^	<0.001
Resp support type, n (%)					
CPAP	115 (58%)	116 (38%)^b^	94 (31%)^b^	14 (6%)^b, c^	<0.001
Conventional	26 (13%)	30 (10%)	15 (5%)^b^	5 (2%)^b, c^	<0.001
High Flow (2+ lpm)	8 (4%)	11 (4%)	13 (4%)	1 (<1%)	0.062
Low Flow (<2 lpm)	48 (24%)	46 (15%)	44 (15%)^b^	0 (0%)^b, c^	<0.001
Duration of respiratory support					
O2 days (Mean ± SD)	2.4 ± 2.0	2.2 ± 1.9	2.2 ± 1.7	2.2 ± 2.0	0.763
O2 days Median (IQR), Range	2 (2), 1–10	2 (2), 1–15	1 (2), 1–8	1 (2), 1–10	0.649
CPAP days (Mean ± SD)	2.8 ± 1.9	2.3 ± 1.5	2.3 ± 1.4	2.2 ± 1.3	0.089
CPAP days Median (IQR), Range	2 (2), 1–12	2 (2), 1–8	2 (2), 1–7	2 (2), 1–5	0.183
MV days (Mean ± SD)	1.4 ± 0.6	1.6 ± 1.0	2.0 ± 1.5	2.0 ± 1.0	0.271
MV days Median (IQR), Range	1 (1), 1–3	1 (1), 1–5	1 (2), 1–6	2 (2), 1–3	0.502
RDS, *n* (%)	40 (20%)	43 (14%)	25 (8%)^b^	21 (9%)^b^	<0.001
BLES, *n* (%)	13 (7%)	17 (6%)	6 (2%)	3 (1%)^b^	0.005
Apnea/Bradycardia, *n* (%)	39 (20%)	22 (7%)^b^	9 (3%)^b^	0 (0%)^b, c^	<0.001
LOS (days)		^b^	^b, c^	^b, c, d^	
Mean ± SD	24 ± 9	17 ± 7	13 ± 6	11 ± 7	<0.001
Median (IQR), Range	22 (10), 12–68	16 (8), 7–47	12 (6), 4–44	10 (8), 3–48	<0.001
Discharge home (days), *n* (%)		^b^	^b, c^	^b, c, d^	
0–10	0 (0%)	35 (11%)	115 (38%)	123 (55%)	<0.001
11–20	83 (42%)	198 (65%)	155 (52%)	86 (38%)	
21–30	78 (39%)	52 (17%)	25 (8%)	9 (4%)	
>30	37 (19%)	21 (7%)	4 (1%)	6 (3%)	

*ART* assisted reproductive technologies, *SGA* small for gestational age, *CPAP* continuous pathway airway pressure, *RDS* respiratory distress syndrome, *MV* mechanical ventilation, *BLES* bovine lipid extract surfactant, *LOS* length of stay.

^a^Chi-square or Fisher’s exact test for categorical variables, ANOVA or Kruskal–Wallis test for continuous variables.

^b^Significantly different than 33 weeks.

^c^Significantly different than 34 weeks.

^d^Significantly different than 35 weeks.

### Infant characteristics

Although BW increased with increasing PMA, no statistically significant difference was observed in infants born between 35- and 36-weeks PMA. Furthermore, a higher proportion of infants were SGA at 36 weeks (28%) compared with the infants born at 33 (14%), 34 (10%) and 35 (8%) weeks PMA (*P* < 0.001; Table 1).

#### Respiratory morbidity

The type and duration of respiratory support (%, Mean ± SD, and Median, IQR and range in days) are detailed in Table 1. Sixty three percent of the infants born at 33-weeks PMA received some form of respiratory support compared with 46%, 39% and 7% at 34-, 35- and 36-weeks, respectively (*P* < 0.001; Table 1). A modest decrease in any form of respiratory support was observed between 33- and 34–35 weeks followed by a sharp decline (39% vs. 7%) at 36 weeks. A larger proportion of infants born at 33 weeks were diagnosed with RDS (20%) vs. those born at 34 (14%), 35 (8%) and 36 (9%) weeks (*P* < 0.001). Similarly, a higher percentage (13%) of the infants were intubated and mechanically ventilated at 33 weeks compared with 2% at 36 weeks PMA (*P* < 0.001). A similar pattern was observed in the administration of exogenous surfactant (*P* = 0.005; Table 1). Higher rates of apnea and/or bradycardia were observed in infants born at 33 weeks (20%), compared with 7%, 3%, and 0% at 34-, 35-, and 36-weeks PMA, respectively (*P* < 0.001).

We observed no difference in infants’ sex, umbilical arterial cord pH, SNAP II, SNAP-PE, PDA, IVH, pneumothorax or pulmonary hypertension across all PMAs (Supplementary Table S1).

#### Length of stay (LOS)

**Fig. 2:**
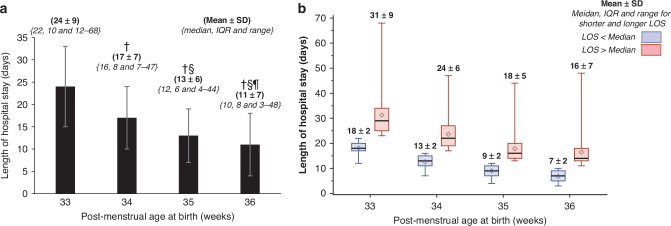
Overall and stratified length of hospital stay. **a**. Overall length of hospital stay (LOS) as Mean ± SD, and M*edian, IQR and Range*. The LOS was 24 ± 9, 17 ± 7, 13 ± 6 and 11 ± 7 days (Mean ± SD in **bold**) at 33-, 34-, 35- and 36-weeks PMA, respectively. The Median and (IQR and range in *italics*) of the LOS were 22 (10; 12–68), 16 (8; 7–47), 12 (6; 4–44) and 10 (8; 3–48) days at the corresponding Post-menstrual Age (PMAs). ^**†**^*P* < 0.05 compared with 33 weeks. ^**§**^*P* < 0.05 compared with 34 weeks. ^¶^*P* < 0.05 compared with 35 weeks. **b**. Stratified by shorter and longer than Median length of hospital stay (LOS) and by post-menstrual Age (PMAs The data points are illustrated as Mean ± SD (**bold**) and Median, IQR and Range (*italicized*) for shorter and longer than Median LOS subgroups at 33-, 34-, 35- and 36-weeks PMA (*P* values not applicable).

The Mean ± SD for the LOS were 24 ± 9, 17 ± 7, 13 ± 6 and 11 ± 7 days at 33-, 34-, 35- and 36-weeks PMA, respectively (*P* < 0.001; Fig. 2a). The Median and (IQR and range) of the LOS were 22 (10; 12–68), 16 (8; 7–47), 12 (6; 4–44) and 10 (8; 3–48) days at the corresponding PMAs (*P* < 0.001; Fig. 2a). The Mean ± SD, and Median, IQR and Range for the LOS, shorter and longer than Median are illustrated in Fig. 2b. The LOS categorized into 0–10, 11–20, 21–30 and >30 days at various PMAs showed almost 20% of the infants born at 33 weeks PMA to remain hospitalized >30 days. Furthermore, at least 45% of the infants admitted at 36 weeks PMA remained in the hospital longer than 10 days after birth (*P* < 0.001; Table 1).

#### Feeding milestones by PMA at birth and respiratory support

The Mean ± SD, Median, IQR and range for achieving gavage-free oral feeds for each PMA are given in Table 2. Most infants received a combination of mothers’ own breast milk, donor human milk and/or formula (44–82%) whereas exclusive use of formula was rare across all PMAs (0% at 33 weeks to 8% at 36 weeks; *P* < 0.001). Although early initiation and accomplishment of oral feeding competence was associated with a shorter LOS, a delay of >3 days between achieving full oral feeds and discharge home was observed in 59%, 43%, 33% and 32% among those born at 33, 34, 35 and 36 weeks PMAs, respectively (*P* < 0.001; Table 2). Furthermore, the lag between achieving gavage-free feeding and being discharged home decreased with increasing PMA (5.7 ± 4.8 at 33 vs. 3.9 ± 4.4 days at 36 weeks, *P* < 0.001). Provision of any form of respiratory support delayed achievement of full oral feeds except at 36 weeks, (*P* ≤ 0.02) indicating an interaction between respiratory support, achievement of gavage-free feeds and LOS. The effect of various forms of respiratory support on achieving full oral feeds is detailed in Table 2.

**Table 2:** Feeding data by post menstrual age (PMA) and in relation to the type of respiratory support.

Feeding Data Summary by Post Menstrual Age at Delivery									
Variable	33 weeks PMA		34 weeks PMA		35 weeks PMA		36 weeks PMA		*p*-value^a^
	*n* = 198		*n* = 306		*n* = 299		*n* = 224		
Type of feed			^b^		^b, c^		^b^		
EBM (+/- DHM) + Formula	87 (44%)		212 (69%)		246 (82%)		167 (75%)		<0.001
EBM (+/-DHM)	111 (56%)		79 (26%)		43 (14%)		39 (17%)		
Formula	0 (0%)		15 (5%)		10 (3%)		18 (8%)		
First feed, post natal day					^b^				
1	139 (70%)		240 (78%)		244 (82%)		166 (74%)		0.018
>1	59 (30%)		66 (22%)		55 (18%)		58 (26%)		
Start of oral feeds, post natal day			^b^		^b^		^b^		
1	87 (44%)		180 (59%)		196 (66%)		141 (63%)		<0.001
2	51 (26%)		80 (26%)		71 (24%)		55 (25%)		
>2	60 (30%)		46 (15%)		32 (11%)		28 (13%)		
Achieving oral feeds PND, days			^b^		^b, c^		^b, c^		
Mean (SD)	18.7 ± 9.6		12.8 ± 6.8		9.4 ± 5.2		7.3 ± 4.5		<0.001
Median (IQR), Range	17 (11), 5–63		11 (8), 1–44		9(6), 1–44		7 (6), 1–24		<0.001
Days between Achieving Oral Feeds and Discharge, days			^b^		^b, c^		^b^		
Mean (SD)	5.7 ± 4.8		4.5 ± 4.1		3.5 ± 3.1		3.9 ± 4.4		<0.001
Median (IQR), range	5 (6), 0–39		3 (3), 0–27		3 (2), 0–27		3 (2), 0–33		<0.001
Days between achieving oral feeds and discharge, *n* (%)			^b^		^b, c^		^b, c^		
0-3	82 (41%)		173 (57%)		201 (67%)		152 (68%)		<0.001
>3	116 (59%)		133 (43%)		98 (33%)		72 (32%)		

Number of postnatal days (Median and IQR) to achieve gavage-free oral feeds in relation to the type of respiratory support									
Variable		33 weeks PMA		34 weeks PMA		35 weeks PMA		36 weeks PMA	
		*n* = 198		*n* = 306		*n* = 299		*n* = 224	
Any respiratory support		17.5 (11)		12 (7)		9 (5)		9 (7.5)	
No respiratory support		16 (8)		11 (7)		7 (5)		7 (6)	
*p*-value^e^		0.019		0.021		<0.001		0.198	
CPAP		18 (11)		13 (7.5)		9 (5)		7 (7)	
No CPAP		16 (9)		11 (8)		8 (5)		7 (6)	
*p*-value^e^		0.021		0.002		0.013		0.459	
Conventional		19 (7)		11 (8)		11 (5)		^f^	
No conventional		16 (11)		11 (8)		8 (5)			
*p*-value^e^		0.059		0.868		0.027			
High nasal flow (2+ lpm)		26 (27)		14 (20)		12 (5)		^f^	
No high nasal flow		16.5 (10)		11 (8)		8 (5)			
*p*-value^e^		0.020		0.117		0.003			
Low nasal flow (<2 lpm)		18.5 (11.5)		13 (10)		10 (4)		^f^	
No low nasal flow		16 (10)		11 (7)		8 (5)			
*p*-value^e^		0.125		0.162		0.024			

*EBM* expressed breast milk, *DHM* donor human milk, *PND* post natal day.

^a^Chi-square or Fisher’s exact test for categorical variables and ANOVA for continuous variables.

^b^Significantly different than 33 weeks.

^c^Significantly different than 34 weeks.

^d^Significantly different than 35 weeks.

^e^Mann–Whitney U test.

^f^Not reported as number of infants on support is <5.

#### Determinants of the short and long LOS

Maternal and infant characteristics associated with the shorter (LOS below the Median) and longer (LOS above the Median) for each PMA are given in Table 3. Multiple births (twins and triplets) at 33 weeks PMA were associated with longer LOS (*P* < 0.001; Table 3) while such relationship was not observed at 34-, 35- or 36-weeks PMA. Birth weight, even within a specific PMA, was associated with longer than Median LOS across 33–35 weeks PMA (*P* ≤ 0.004). Similar association was observed for SGA infants (*P* ≤ 0.04). However, the proportion of SGA with longer LOS had an inverse relationship with PMA, decreasing with increasing PMA until 35 weeks PMA. For example, at 33 weeks, the proportion of SGA infants in the longer LOS group was sixfold that of the shorter LOS group compared with 4.5 and 2.4-fold at 34- and 35-weeks PMA, respectively (Table 3). While the proportion of outborn infants was similar in the longer LOS and shorter LOS groups at 33 weeks (*P* = 0.11), the proportion of outborn infants in the longer LOS group was significantly higher than in the shorter LOS group for infants at 34–36 weeks (*P* ≤ 0.001). Any respiratory support including administration of CPAP was associated with longer hospital stay at 33- and 35-weeks PMA (*P* ≤ 0.027). Similarly, the frequency of RDS was higher in for those with a longer LOS at 33- and 36-weeks PMA (*P* ≤ 0.006). Initiation of oral feeding on PND one was associated with shorter length of stay among infants born at 35- and 36-weeks PMA (*P* ≤ 0.001). Achieving gavage-free oral feeds earlier was consistently the main determinant of early vs. late discharge home across all PMAs. Furthermore, it took almost twice as long for infants to achieve gavage-free feeds in those who stayed longer than the Median LOS across all PMAs (*P* < 0.001; Table 4).

**Table 3:** Maternal and infant characteristics in relation to shorter and longer than median length of stay (days).

	33 weeks PMA (*n* = 198)			34 weeks PMA (*n* = 306)			35 weeks PMA (n = 299)			36 weeks PMA (*n* = 224)		
	LOS ≤ 22 d (*n* = 103)	LOS > 22 d (*n* = 95)	p-value	LOS ≤ 16 d (*n* = 175)	LOS > 16 d (*n* = 131)	*p*-value	LOS ≤ 12 d (*n* = 171)	LOS > 12 d (*n* = 128)	*p*-value	LOS ≤ 10 d (*n* = 123)	LOS > 10 d (*n* = 101)	*p*-value
Maternal characteristics												
Age, mean (SD)	33 (5)	32 (5)	0.120	34 (5)	33 (6)	0.203	33 (5)	32 (5)	0.060	33 (5)	33 (5)	0.316
ART, *n* (%)	17 (16%)	19 (20%)	0.524	37 (21%)	25 (19%)	0.658	20 (12%)	21 (16%)	0.241	16 (13%)	10 (10%)	0.470
Multiple Birth, *n* (%)	28 (27%)	48 (51%)	<0.001	48 (27%)	46 (35%)	0.149	53 (31%)	50 (39%)	0.146	38 (31%)	27 (27%)	0.495
Hypertension/PE, *n* (%)	26/102 (25%)	19 (20%)	0.359	32 (18%)	38 (29%)	0.027	39 (23%)	39 (30%)	0.134	33 (27%)	32 (32%)	0.426
Gestational hypertension, *n* (%)	26/102 (25%)	19 (20%)	0.359	31 (18%)	38 (29%)	0.019	37 (22%)	38 (30%)	0.112	31 (25%)	29 (29%)	0.555
Antenatal steroids, *n* (%)	89/102 (87%)	82/94 (87%)	0.997	83/171 (49%)	67/130 (52%)	0.606	38 (22%)	37/125 (30%)	0.150	18 (15%)	14 (14%)	0.869
Antenatal steroids full course (2 or more doses), *n* (%)	56/102 (55%)	48/94 (51%)	0.591	50/169 (30%)	49/129 (38%)	0.127	23 (13%)	26/125 (21%)	0.093	15 (12%)	10 (10%)	0.587
Infant characteristics												
Birth weight, mean (SD)	2046 ± 325	1794 ± 392	<0.001	2282 ± 357	2055 ± 383	<0.001	2519 ± 406	2376 ± 426	0.004	2542 ± 469	2523 ± 606	0.798
Male, *n* (%)	53 (51%)	41 (43%)	0.243	95 (54%)	64 (49%)	0.347	86 (50%)	64 (50%)	0.960	72 (59%)	60 (59%)	0.895
SGA, *n* (%)	4 (4%)	23 (24%)	<0.001	7 (4%)	23 (18%)	<0.001	9 (5%)	15 (12%)	0.042	29 (24%)	33 (33%)	0.130
Outborn, *n* (%)	7 (7%)	13 (14%)	0.108	4 (2%)	17 (13%)	<0.001	1 (1%)	10 (8%)	0.001	3 (2%)	15 (15%)	<0.001
Resuscitation, *n* (%)	22 (21%)	11 (12%)	0.065	50 (29%)	36 (27%)	0.834	68 (40%)	48 (38%)	0.795	48 (39%)	34 (34%)	0.407
Any respiratory support (Vent/CPAP/NP), *n* (%)	56 (54%)	68 (72%)	0.012	75 (43%)	66 (50%)	0.191	56 (33%)	62 (48%)	0.006	8 (7%)	8 (8%)	0.682
Respiratory support type *n* (%)												
CPAP	51 (50%)	64 (67%)	0.011	60 (34%)	56 (43%)	0.131	45 (26%)	49 (38%)	0.027	8 (7%)	6 (6%)	0.862
Conventional	8 (8%)	18 (19%)	0.020	16 (9%)	14 (11%)	0.653	5 (3%)	10 (8%)	0.055	1 (1%)	4 (4%)	0.178
High Nasal Flow (2+ lpm)	2 (2%)	6 (6%)	0.157	4 (2%)	7 (5%)	0.215	3 (2%)	10 (8%)	0.011	0 (0%)	1 (1%)	0.451
Low Nasal Flow (<2 lpm)	19 (18%)	29 (31%)	0.048	22 (13%)	24 (18%)	0.164	20 (12%)	24 (19%)	0.088	0 (0%)	0 (0%)	n/a
RDS, *n* (%)	13 (13%)	27 (28%)	0.006	20 (11%)	23 (18%)	0.127	10 (6%)	15 (12%)	0.070	5 (4%)	16 (16%)	0.003
BLES, *n* (%)	3 (3%)	10 (11%)	0.031	8 (5%)	9 (7%)	0.385	2 (1%)	4 (3%)	0.408	1 (1%)	2 (2%)	0.590
Apnea/Bradycardia, *n* (%)	13 (13%)	26 (27%)	0.009	9 (5%)	13 (10%)	0.109	4 (2%)	5 (4%)	0.504	0 (0%)	0 (0%)	n/a

*ART* assisted reproductive technology, *SGA* small for gestational age, *CPAP* continuous pathway airway pressure, *RDS* respiratory distress syndrome, *BLES* bovine lipid extract surfactant, *LOS* length of stay.

**Table 4:** Infant feeding data in relation to shorter and longer than median length of stay (LOS in days).

	33 weeks PMA (*n* = 198)			34 weeks PMA (*n* = 306)			35 weeks PMA (*n* = 299)			36 weeks PMA (n = 224)		
	LOS ≤ 22 d (*n* = 103)	LOS > 22 d (*n* = 95)	*p*-value	LOS ≤ 16 d (*n* = 175)	LOS > 16 d (*n* = 131)	*p*-value	LOS ≤ 12 d (*n* = 171)	LOS > 12 d (*n* = 128)	*p*-value	LOS ≤ 10 d (*n* = 123)	LOS > 10 d (*n* = 101)	*p*-value
First feed PND, *n* (%)												
1	76 (74%)	63 (66%)	0.251	145 (83%)	95 (73%)	0.030	146 (85%)	98 (77%)	0.052	101 (82%)	65 (64%)	0.003
>1	27 (26%)	32 (34%)		30 (17%)	36 (27%)		25 (15%)	30 (23%)		22 (18%)	36 (36%)	
Start of oral feeds PND												
1	51 (50%)	36 (38%)	0.187	112 (64%)	68 (52%)	0.079	125 (73%)	71 (55%)	0.001	91 (74%)	50 (50%)	<0.001
2	26 (25%)	25 (26%)		42 (24%)	38 (29%)		36 (21%)	35 (27%)		26 (21%)	29 (29%)	
>2	26 (25%)	34 (36%)		21 (12%)	25 (19%)		10 (6%)	22 (17%)		6 (5%)	22 (22%)	
Achieving oral feeds PND												
Median (IQR), Range	13 (6), 5–22	23 (12), 6–63	<0.001	9 (4), 1–16	16 (8), 4–44	<0.001	7 (3), 1–11	12 (5), 3–44	<0.001	4 (5), 1–10	10 (5), 1–24	<0.001
Days between achieving oral feeds and discharge												
Median (IQR), range	4 (5), 0–13	5 (6), 1–39	0.271	3 (2), 0–13	4 (7), 0–27	<0.001	2 (2), 0–10	4 (4), 0–27	<0.001	2 (2), 0–6	4 (5), 1–33	<0.001
Days between achieving oral feeds and discharge, *n* (%)												
0–3	43 (42%)	39 (41%)	0.921	114 (65%)	59 (45%)	<0.001	142 (83%)	59 (46%)	<0.001	105 (85%)	47 (47%)	<0.001
>3	60 (58%)	56 (59%)		61 (35%)	72 (55%)		29 (17%)	69 (54%)		18 (15%)	54 (53%)	

*PND* postnatal day, *LOS* length of hospital stay.

#### Logistic regression models predicting the longer LOS (Table 5)

The candidate variables, odds ratios [OR and (95% Confidence Intervals)] for maternal and infant characteristics associated with both shorter and longer than Median LOS for each PMA (33 through 36 weeks) are detailed in Table 5. After excluding the variables in the causal pathways, multiple births [2.79 (1.4–5.5)], SGA status [4.3 (1.1–17.4)], RDS [5.1 (2.2–12)], lower maternal age [0.9 (0.87–0.98)]and lower BW [0.99 (0.99–1.0)] were associated with longer LOS at 33 weeks PMA. At 34 weeks, outborn infants [17.7 (4.2–74.5)] and lower BW [0.99 (0.99–0.99)] were associated with increased odds of longer LOS. For infants born at 35 weeks, outborn infants [13.4 (1.7–108)], those with PDA [15.5 (1.9–126.5)] and lower BW [0.99 (0.99–1.0)] had a longer LOS, while at 36 weeks, the outborn infants had increased odds [6.5 (1.8–23.4)] of longer LOS.

**Table 5:** Logistic regression models predicting longer lengths of stay at 33 through 36 weeks of post-menstrual age (defined as greater than the median length of stay).

Analysis of maximum likelihood estimates						
Parameter	DF	Estimate	Standard error	Wald chi-square	Pr > ChiSq	Odds ratio estimate (95% C.I.)
Number of births (multiple vs. singleton)	1	1.0247	0.3513	8.5087	0.0035	2.786 (1.400–5.546)
SGA	1	1.4678	0.7098	4.2755	0.0387	4.340 (1.079–17.445)
RDS	1	1.6332	0.4365	13.9996	0.0002	5.120 (2.176–12.045)
MaternalAge	1	–0.0777	0.0334	5.4217	0.0199	0.925 (0.867–0.988)
BrthWght	1	–0.00169	0.000641	6.9277	0.0085	0.998 (0.997–1.000)
Birthplace (outborn vs. inborn)	1	2.8735	0.7333	15.3536	<.0001	17.699 (4.205–74.503)
BrthWght	1	–0.00216	0.000386	31.2277	<.0001	0.998 (0.997–0.999)
Birthplace (outborn vs. inborn)	1	2.5970	1.0641	5.9571	0.0147	13.424 (1.668–108.046)
PDA	1	2.7380	1.0725	6.5170	0.0107	15.456 (1.889–126.482)
BrthWght	1	–0.00091	0.000320	8.1266	0.0044	0.999 (0.998–1.000)
Intercept	1	–0.3331	0.1413	5.5600	0.0184	
Birth place (outborn vs. inborn)	1	1.8735	0.6517	8.2647	0.0040	6.511 (1.815–23.355)

Logistic regression models predicting longer lengths of stay (defined as greater than the median length of stay) for 33 weeks:

Candidate variables include maternal age, gestational diabetes, systemic antibiotics, multiple birth, birth weight, SGA, resuscitation, SNAP PE score, outborn, RDS, pneumothorax, apnea/bradycardia.

Logistic regression models predicting longer lengths of stay (defined as greater than the median length of stay) for 34 weeks:

Candidate variables include maternal age, gestational hypertension, antenatal steroids full course, multiple birth, birth weight, SGA, outborn, RDS, pneumothorax, apnea/bradycardia, non-Calgary discharge hospital.

Logistic regression models predicting longer lengths of stay (defined as greater than the median length of stay) for 35 weeks:

Candidate variables include maternal age, gestational hypertension, ART, antenatal steroids full course, cesarean delivery, multiple birth, birth weight, SGA, outborn, PDA, RDS, pneumothorax, PPHN.

Logistic regression models predicting longer lengths of stay (defined as greater than the median length of stay) for 36 weeks:

Candidate variables include systemic antibiotics, SGA, SNAP II, outborn, PDA, RDS, non-Calgary discharge hospital.

*BrtWght* birth weight, *CPAP* continuous positive airway pressure, *PDA* patent ductus arteriosus, *PMA* post-menstrual age, *RDS* respiratory distress syndrome, *SGA* small for gestational age, SNAP PE score score for Neonatal Acute Physiology (SNAP II) and SNAP with perinatal extension.

## Discussion

Almost every day in a neonatal intensive care setting, parents ask neonatal health care providers about the anticipated postnatal course and the expected date of discharge home. It is difficult to answer these questions precisely without the availability of PMA-specific morbidities. This study significantly advances the field by providing seminal data on several inter-related variables and outcomes, potentially applicable to several million preterm infants born globally each year between 33- and 36-weeks PMA. These data will inform the pediatricians and subspecialists including neonatal clinicians about the anticipated neonatal course and play an important role for counseling the parents with additional PMA-specific precision.

We took a novel approach of stratifying infants around the shorter and longer than Median LOS. Such strategy identified several variables associated with prolonged LOS such as BW even within a given PMA, place of birth, SGA status, respiratory support, RDS, initiation of first overall and oral feeds and achievement of gavage-free feeds. Furthermore, we observed interactions not only between respiratory support and gavage-free feeds but also between respiratory support and LOS, implying that delayed gavage feeding, related to delayed discharge, in turn, might be due to other co-morbidities such as respiratory insufficiency and invasive/non-invasive respiratory support. For example, this study provides incidences of various morbidities such as non-invasive and invasive respiratory support, role of SGA status and birthweight, and impact of being transferred from level II to level III NICU (for more intensive level of care) at various PMAs. Furthermore, study can inform the parents of an approximate cut-off in birth weight, presence of SGA status and provision of any respiratory support being associated with longer LOS. The study may also validate clinicians’ impression based on their experience, which variables play an important role in determining the LOS.

Each PMA presents unique challenges and opportunities. Thus, our results demonstrate that grouping various PMAs^19,32–34^ into single entities and/or investigating individual morbidities^19,20,33,39–41,58^ in isolation do not represent the true burden of disease in preterm infants at a given PMA, and has the potential for providing imprecise information to the clinicians and parents.

Due to heterogeneity, PMA rather than BW has been identified as the determinant of both short- and long-term outcomes.^59–62^ We demonstrate that birthweight is a significant variable in determining the LOS except those born at 36 weeks PMA, which could reflect better competency in oral feeding, mature thermoregulation, and less occurrence of apneas, bradycardias and oxygen desaturation episodes. One study reported that infants weighing >2 kg were discharged home earlier than those weighing <2 kg, however, the infants born at 30–34 weeks PMA were not stratified according to the birth weight.^10^ At a much younger PMA (22–25 weeks), higher BW even within a given PMA was associated with 2–4-fold less mortality and morbidity.^63^ Our study provides an approximate birth weight cut-off, associated with longer or shorter than Median length of stay at 33–35 weeks PMA suggesting that birth weight at any PMA is a critical variable that needs to be considered when counseling parents or planning home discharge. This PMA-specific information may be used not only for informing the parents but also for projecting bed capacity and ensuring adequate bedside coverage.^14,17^

Past studies have reported a wide variability (8.5%^19^ to 83%^32^) in the use of respiratory support, likely reflecting population heterogeneity.^11,64^ Over the past decade, an increasing number of preterm infants with respiratory insufficiency are being managed using non-invasive modes of ventilation.^39,65–72^ Direct comparisons with previous studies cannot be made as infants were either grouped together, comparisons made with term infants,^34,73^ and/or the incidence, type and duration of respiratory support were either not reported,^31^ specified and/or important clinical variables including birth weight were not captured.^34,36^ We provide evidence that the incidence and type of respiratory support is at least 5–6-fold higher in 34- and 35-weeks PMA infants vs. those born at 36-weeks. Such variability in the traditionally combined late preterm group would mask the true prevalence. Hence, in the era of increasing use of non-invasive respiratory support,^66,69,70^ our study represents the current trends of managing respiratory insufficiency in preterm infants.^39^

Establishment of oral feeding, a repertoire of suck-swallow-breathe coordination is one of the most complex yet vastly underappreciated motor function in preterm infants.^53,58,74–78^ In our study, we have investigated the multi-stage process of oral feeding and its interaction with respiratory insufficiency, as reaching full oral feeds is one of the major criteria for discharge home. We demonstrate that despite reaching full oral feeds, more immature infants experienced a longer hospital stay. Such delay likely represents lower PMA infants, who received higher respiratory support and/or needing apnea-free observation while in-patients. To an extent, our study supports previous work showing the relationship between early start of feeds and discharge home. However, we provide further evidence that such relationship was not observed at the youngest PMA and represents slower development of suck-swallow-breathe coordination,^53,75,76^ which might have been amplified due to the need for both invasive and non-invasive respiratory support.

As pointed out previously,^12^ there is an assumption (“rule of thumb”) that moderate and late preterm babies may be discharged home around 36 weeks PMA. Our results demonstrate that such assumption is inaccurate as each PMA offers unique challenges. We show that as many as 58% and over 35% of the infants born at 33 and 34 weeks, respectively, may still be in-patients by 36 weeks PMA. Some of the interesting significant variables included longer LOS in infants of younger mothers, multiple births but only at 33 weeks and lack of the effect of infant’s sex (male) on LOS, which was previously shown to be a significant variable,^31^ likely due to much larger sample size. The longer LOS among multiples (twins and triplets) at the youngest PMA in our study might be due to lower BWs, higher needs for respiratory support, recurring apneas along with a delayed achievement of full oral feeds. The longer LOS among multiples has previously been reported in previous study but in different cohort style.^31^ The data on the LOS need to be interpreted with caution as it varies on the level of the NICUs (level 2 vs. 3). Furthermore, infants’ stay in a mother-baby unit, does not equate to discharge home and simply means not admitted to the NICU.^48^ Moderate and late preterm infants represent the fastest growing group of preterm infants. Such rapid increase has put high demands on bed capacity,^14^ and recruitment and retention of health care personnel. Although the financial burden per infant is less in moderate and late preterm infants (32–36 weeks PMA) compared with the very preterm infant (<28 weeks PMA), due to the sheer high number of the moderate and late preterm infants (>80% of the total preterm population), the cumulative cost is several-fold higher compared with the very preterm infants.^79^

### Parental needs

In a recent study, parents identified their desire for clearer explanation for the reason for admission, NICU course and discharge planning.^28^ Achievement of these parental needs will be challenging if not impossible without the availability of PMA-specific morbidities and variability in the LOS even within a given PMA as demonstrated in our study. Hence, our study fills a an important knowledge-gap not only for clinicians but more so for parental counseling as it identifies the importance of birth weight, place of birth, multiple births and provision of any respiratory support to the LOS. These variables can be used to inform parents if their infant would have shorter or longer than Median LOS.

### Strengths and limitations

The major strengths of our study include: (1) analysis of the complex interaction between various morbidities at a given PMA while excluding the variables in the causal pathways, (2) PMA-specific incidence, type, and duration of respiratory support, (3) detailed analyses including visual review of enteral and oral feeding milestones of 1027 infants, (4) the novel approach of identifying variables associated with the prolonged LOS by stratifying the infants with longer and shorter than Median length of stay, (5) availability of the complete dataset for each infant from admission till discharge home, (6) contemporary knowledge-translation for general pediatricians, neonatologists and bedside physicians and (7) generalizability of the information as Foothills Medical Centre serves not only as a tertiary care regional perinatal center but also level I and II Center for the northwest quadrant of the city of 1.6 million inhabitants. This is important as nature of care may differ between level II and III NICUs.^80,81^ The limitations of our study include not including 32 weeks PMA, non-stratification of results based on the SGA status of the infants, etiology of respiratory distress, and time to regain birth weight especially in relation to hyperbilirubinemia and phototherapy, which may be associated with slower oral-feeding skills in a preterm animal model.^82^ On the other hand, although healthy term infants with hyperbilirubinemia may appear disinterested in oral feeding, their milk intake was not adversely affected.^83^ These differences might be related to species or gestational age differences.^82,83^ Some of the limitations identified in our study have previously been addressed.^42,84^ Finally, due to the single payer system in Alberta and in the setting of Canadian universal health care system, we could not quantify the impact of various payer systems on neonatal care practices.^31^

## Conclusions

This study addresses knowledge gaps and provides contemporary data that contribute to a better understanding of the neonatal morbidities in this fastest-growing population of preterm infants. To our knowledge, our study provides the most comprehensive data on PMA-specific incidence, type, and duration of respiratory support, achievement of feeding milestones, and stratified LOS in a single cohort of infants born between 33 and 36 weeks PMAs. We demonstrate significant inter-dependence between PMA, respiratory support, several infant characteristics, and delayed achievement of full oral feeds. Availability of contemporary data may assist health care providers to inform parents of anticipated postnatal course and an approximate LOS at various PMAs.^14,16,17,28,85,86^ We and others have demonstrated that the morbidities increase in an inverse relationship to the PMA at birth and an increase of PMA even by one week at birth significantly reduces the morbidities and economic costs, hence a concerted effort needs to be made to decrease the ever-increasing rates of late preterm infants at birth.^79^

## Supplementary information

Supplementary Information

## Data Availability

The corresponding author will provide the original data supporting the results and conclusion of this article upon reasonable request and after obtaining IRB approval.
