# Supplementary material for: Respiratory insufficiency, feeding issues and length of stay in 33–36 weeks post-menstrual age infants
Source: Pediatr Res. 2025 Sep 27;99(4):1504–16. doi: 10.1038/s41390-025-04411-4 (PMC13102693; doi:10.1038/s41390-025-04411-4)
Supplement: Supplementary file 1 — Supplementary Information [file 41390_2025_4411_MOESM1_ESM.pdf]

Supplemental Table S1. Non-Significant Maternal and Infant Characteristics by Postmenstrual Age

	Post Menstrual Age (PMA)				
**Maternal Characteristics**	33 weeks n=198	34 weeks n=306	35 weeks n=299	36 weeks n=224	p-value*
Diabetes Mellitus, n (%)	37 (19%)	60 (20%)	55 (18%)	52 (23%)	0.538
Gestational Diabetes, n (%)	37 (19%)	60 (20%)	55 (18%)	52 (23%)	0.538
Hypertension/PE, n (%)	45/197 (23%)	70 (23%)	78 (26%)	65 (29%)	0.345
Gestational Hypertension, n (%)	45/197 (23%)	69 (23%)	75 (25%)	60 (27%)	0.662
Pre-existing Hypertension, n (%)	0/197 (0%)	1 (>1%)	3 (1%)	5 (2%)	0.058
Cesarean Delivery, n (%)	123/197 (62%)	169/305 (55%)	155/298 (52%)	132 (59%)	0.114
Maternal Fever, n (%)	7/194 (4%)	7/304 (2%)	7/297 (2%)	2/221 (1%)	0.328
Systemic Antibiotics, n (%)	105/197 (53%)	171/303 (56%)	148/296 (50%)	103/223 (46%)	0.113
Multiple Birth, n (%)	76 (38%)	94 (31%)	103 (34%)	65 (29%)	0.157
History of Drug Use	4 (2%)	10 (3%)	9 (3%)	6 (3%)	0.864
Cigarettes/Smoking	11 (6%)	18 (6%)	15 (5%)	13 (6%)	0.968
**Infant Characteristics**					
Arterial cord pH, mean (SD)	7.25 (0.09)<br>Range 6.94-7.52<br>(missing=58)	7.26 (0.08)<br>Range 6.99-7.44<br>(missing=92)	7.24 (0.08)<br>Range 6.85-7.41<br>(missing=72)	7.24 (0.09)<br>Range 6.83-7.43<br>(missing=61)	0.060
SNAP II, n (%)<br>0<br>1-10<br>>10	 115 (58%)<br>64 (32%)<br>19 (10%)	 183 (60%)<br>92 (30%)<br>31 (10%)	 172 (58%)<br>91 (30%)<br>36 (12%)	 143 (64%)<br>67 (30%)<br>14 (6%)	0.457
SNAP PE, n (%)<br>0<br>1-10<br>>10	 101 (51%)<br>57 (29%)<br>40 (20%)	 169 (55%)<br>85 (28%)<br>52 (17%)	 165 (55%)<br>83 (28%)<br>51 (17%)	 124 (55%)<br>59 (26%)<br>41 (18%)	0.951
Five minute APGAR <7, n(%)	14 (7%)	17/303 (6%)	18/298 (6%)	26 (12%)	0.044#
PDA, n (%)	10 (5%)	6/305 (2%)	10 (3%)	7 (3%)	0.297
IVH, n (%)	2 (1%)	1 (<1%)	2 (1%)	1 (<1%)	0.743
Pneumothorax, n (%)	5 (3%)	14 (5%)	7 (2%)	12 (5%)	0.195
PPHN, n (%)	5 (3%)	4 (1%)	5 (2%)	2 (1%)	0.581
Discharge Hospital, n (%)<br>Calgary<br>Non-Calgary	 178 (90%)<br>20 (10%)	 280 (92%)<br>26 (8%)	 285 (95%)<br>14 (5%)	 205 (92%)<br>19 (8%)	0.116

SNAP II - Score for Neonatal Acute Physiology, SNAP PE - Score for Neonatal Acute Physiology with perinatal extension, PDA - Patent Ductus Arteriosus, IVH - Intraventricular Hemorrhage, PPHN - Persistent Pulmonary Hypertension
